# Prevalence and diversity of parasitic bird lice (Insecta: Psocodea) in northeast Arkansas

**DOI:** 10.1016/j.ijppaw.2023.06.007

**Published:** 2023-07-21

**Authors:** Paige J. Brewer, Andrew D. Sweet

**Affiliations:** Department of Biological Sciences, Arkansas State University, Jonesboro, AR, United States

**Keywords:** Phthiraptera, host‐parasite associations, PCR, cox1, EF1‐α

## Abstract

Many groups of parasites lack basic information on biodiversity and host associations, which poses challenges for conservation and understanding the ecological relationships between hosts and their parasites. This gap in knowledge is particularly relevant for parasitic species with obscure lifestyles. Ectoparasitc bird lice (Insecta: Psocodea: Phthiraptera) are a group of parasites that has received a relatively substantial research focus, yet patterns of bird-louse relationships and louse diversity remain understudied in many geographic regions, including in parts of the southeastern United States. In this study, we assessed the diversity, prevalence, abundance, and intensity of lice from live and salvaged birds in northeastern Arkansas. We also focused on the frequency of co-occurrence of lice and symbiotic feather mites. Finally, we used nuclear and mitochondrial genes to assess the phylogenic relationships among the most common genera of lice in our sample. We found a total louse prevalence of 10.57% with the highest prevalence on the Passeriformes families Turdidae, Passerellidae, and Parulidae. We also found the louse genera *Myrsidea* and *Brueelia* to be the most prevalent and abundant in our sample. Additionally, we reported several novel associations among well-studied bird species. We also found that louse phylogenic patterns tend to reflect host taxonomy and/or ecology. Overall, our results provide important insight into the biodiversity, community structure, and host interactions of parasitic lice from North American birds.

## Highlights

• Variation in louse prevalence among several taxonomic groups of hosts.
• Low ectosymbiont co-occurrences of mites and lice.
• Phylogenetic patterns of *Myrsidea* and *Brueelia* reflect host taxonomy or host ecology.
• Several novel associations not previously recorded among even common and well-researched host species.

## Graphical abstract

**Figure:**
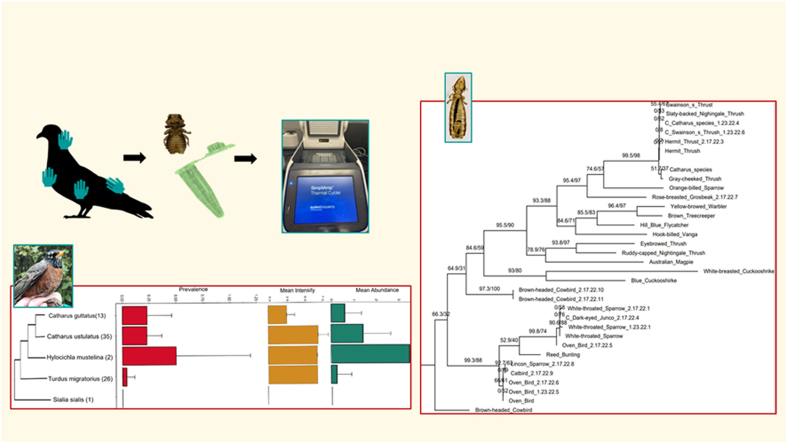

## 1 Introduction

Despite making up approximately 70% of species on earth, parasites are often under-accounted for in most ecosystems (Lafferty et al., 2008; Dougherty et al., 2016). This lack of parasite data can cause a bias in measurements of biodiversity and an incomplete picture of trophic interactions (Hudson et al., 2006). Without a historic baseline of data for groups of parasites, fluctuations in parasite populations are difficult to detect. For example, a significant proportion (5–10%) of parasite species are predicted to go extinct by 2070 due to climate related factors; however, this hypothesis is challenged by the lack of data on population dynamics and biodiversity for many groups of parasites and/or geographic regions (Poulin et al., 2011; Toit et al., 2013; Carlson et al., 2017). The limited dispersal of some parasites decreases effective population sizes and restricts gene flow (Madrid et al., 2020). This can result in parasites being vulnerable to extinction, even when their hosts are not (Madrid et al., 2020).

Birds are host to several well-studied groups of parasites and other symbionts, including ectosymbionts such as feather mites (Acari) and lice (Insecta: Psocodea). Feather mites are small arachnids that live among feathers of birds, primarily in the wing and tail feathers. These mites are generally stationary and most likely have a mutualistic or commensal relationship with their hosts (Bruin et al., 2018). Lice, on the other hand, are parasitic insects that live on different parts of their host's body. Of the approximately 5,000 described species of lice (including mammal and bird lice), approximately 80% are exclusive to bird hosts (Price et al., 2003). Bird lice are grouped into two clades based on their morphology and phylogenetic placement (Price et al., 2003; De Moya et al., 2021). Most lice in the parvorder Ischnocera are true feather specialists that feed on soft downy feathers, whereas lice in the parvorder Amblycera feed on feather, skin, or blood (Nelson and Murray, 1971). Lice are further distinguished into “ecomorphs” based on their morphology and niche specialization. Some louse ecomorphs are adapted to live in specific types of feathers to avoid being removed by their hosts (Bush, 2009; Sweet and Johnson, 2018). For example, lice that primarily occupy their host's head have strong mandibles to prevent removal from blunt forces such as scratching (Johnson et al., 2012). Similarly, “wing” lice primarily live on wing feathers and avoid preening by inserting themselves between the feather barbs (Bush and Clayton, 2006; Sweet and Johnson, 2018). Many louse taxa also have specific relationships with particular groups of hosts, in part due to their limited dispersal abilities (Johnson et al., 2012; Koop et al., 2014; Villa et al., 2018). The majority of bird lice spend their entire 4-8-week life on a single host individual (Bush et al., 2006). Dispersal of lice most often occurs through direct contact with another bird, such as mates or offspring (Bush et al., 2006). However, some lice are capable of dispersing via phoresy on mobile bird parasites such as hippboscid flies (Harbison et al., 2009). The infrequent dispersal of lice to different birds often results in lice which have highly specific relationships with one or a few closely related hosts species (Koop et al., 2014). This high degree of adaptation for specific host species can result in strongly congruent evolutionary relationships between the lice and their hosts, making this system ideal for studying codiversification and ecological processes in host-parasite relationships (Clayton and Walther, 2001; Clayton and Johnson, 2003; Sweet and Johnson, 2016). Furthermore, bird and louse interactions allow us to gain insights on drivers of parasite prevalence, dispersal abilities, and biodiversity (Clayton and Walther, 2001; Sweet and Johnson, 2016).

The prevalence and intensity of bird lice varies across different host taxa and geographic regions (Paterson et al., 1999; Soto-Patiño et al., 2018). This variation can be linked to several factors including host health, host morphology, host habitat, and behavior (Clayton and Walther, 2001). Birds preen and groom themselves to decrease lice loads. Birds in poor health often have higher lice loads, perhaps due to reduced time devoted to removing lice (Ash, 1960; Cochrane, 2011). Furthermore, louse prevalence and intensity often vary relative to morphology such as host body size and bill shape (Chu et al., 2019). Additionally, the prevalence of bird lice is predicted to be dependent on the host's habitat (Carlson et al., 2017). Because lice acquire water from the atmosphere, they often have a higher abundance in humid climates (Carrillo et al., 2007). For example, a study of bird lice from drier regions in Texas found approximately 13% prevalence across different bird species (Pistone et al., 2021), whereas Neotropical birds often have more than 50% prevalence (Clayton et al., 1992; Clayton and Walther, 2001). Prevalence is also shaped by host phenology and behavior such as mating and brooding seasons (Sychra et al., 2011). In addition, birds with higher flocking frequencies tend to have higher louse prevalence than territorial, solitary birds (Tomás et al., 2016). In combination, these factors shed light on why certain host groups may have higher prevalence than others.

Despite the considerable body of research on bird lice, patterns of prevalence and diversity of lice remain unknown for many host groups and geographic regions (Bush, 2004; Carrillo et al., 2007; Bush et al., 2009; Durkin et al., 2015; Gustafsson and Bush, 2020; Pistone et al., 2021). These patterns are overlooked in part due to the obscure lifestyles and cryptic morphology of lice. Louse biodiversity is likely particularly underestimated, which is supported by a steady stream of new species descriptions and host records (Bush et al., 2016; Adly et al., 2019; Madrid et al., 2020). Additionally, not much is known about their relationships with other bird ectosymbionts such as feather mites. The southeast region of the United States, and particularly the state of Arkansas, is one region that has relatively few studies focused on ectosymbionts of birds (Hood and Welch, 1980; McAllister et al., 2019). There have been several surveys of ectoparasites from particular species or group of birds from the region, but no studies have compared prevalence and genetic patterns across broader taxonomic samples (Edgar, 1953; Bush et al., 2009; Garbarino et al., 2013; Pistone et al., 2021).

In this study, we assessed the diversity, prevalence, abundance, and intensity of lice from wild and previously collected birds in northeastern Arkansas. We focused on four questions: (1) Is there variation in louse prevalence and abundance among different groups of birds? (2) What are the most common taxa of bird lice? (3) How frequently do lice and mites co-occur on bird hosts? (4) What is the phylogenetic diversity among lice from various bird groups? We expected to yield a relatively high prevalence of bird lice due to the humid climate in the southern United States. We also expected to discover novel associations between birds and lice due to under sampling in this region. This research provides a contribution for better understanding biodiversity, community structure, and life history of parasites.

## 2 Materials and methods

### 2.1 Lice collection and identification

A total of 680 birds from 30 families and 8 orders were examined for lice and mites (Table 1, Table S1). This included 578 individuals in the order Passeriformes. These birds were sourced from both preserved specimens in the Arkansas State University (A-State) collection and live-captured individuals in northeast Arkansas. Preserved birds were stored individually in plastic bags to prevent ambiguity in host-parasite associations from cross contamination. Collection dates for these birds ranged from 1994 to 2022 with the majority between 2015 and 2021. Most birds were collected from northeast Arkansas, but some (214) were collected near Augustana College in Rock Island, IL, and 14 individuals from Texas, Tennessee, Louisiana, or Missouri. The cause of death for most individuals was window collision however 16 individuals likely died from extreme temperatures during a cold period in Arkansas during February 2021. Most birds were collected within hours after death. Any birds in poor conditions were excluded from the sampling, including birds with evidence of infestation with ants prior to collection, birds with many missing feathers, and/or specimens that were severely degraded. Each specimen was thawed and the feathers robustly ruffled to dislodge the lice onto a white sheet of paper. Ruffling was emphasized in the downy feathers on the chest and flanks of the birds, where lice often congregate when the host is placed in a freezer. The birds were also visually examined for lice and mites. Feather mites are often found between the barbs of wing and tail feathers, so these feathers were carefully examined against a light to check for mites.

**Table 1:** Infestation statistics for chewing lice sampled from different families of avian hosts, including prevalence, mean intensity, mean abundance, and associated 95% confidence intervals.

Order	Family	**Sample size (Host individuals)**	Prevalence	**Prevalence confidence intervals**	Mean Intensity	**Intensity confidence intervals**	Mean abundance	**Abundance confidence** intervals
**Accipitriformes**								
	Accipitridae	1	100.00	0.00	1.00	0.00	1.00	0.00
**Anseriformes**								
	Anatidae	3	33.33	0.53	2.00	0.00	0.67	0.00
**Apodiformes**								
	Trochilidae	28	0.00	0.00	0.00	0.00	0.00	0.00
**Charadriiformes**								
	Scolopacidae	1	100.00	0.00	11.00	0.00	11.00	0.00
**Columbiformes**								
	Columbidae	11	0.00	0.00	0.00	0.00	0.00	0.00
**Gruiformes**								
	Rallidae	7	14.29	0.26	7.00	0.00	1.00	0.00
**Passeriformes**								
	Bombycillidae	16	0.00	0.00	0.00	0.00	0.00	0.00
	Cardinalidae	34	8.82	0.10	2.33	0.51	0.21	0.27
	Certhiidae	6	0.00	0.00	0.00	0.00	0.00	0.00
	Corvidae	4	0.00	0.00	0.00	0.00	0.00	0.00
	Emberizidae	3	0.00	0.00	0.00	0.00	0.00	0.00
	Fringillidae	2	0.00	0.00	0.00	0.00	0.00	0.00
	Hirundinidae	1	100.00	0.00	3.00	0.00	3.00	0.00
	Icteridae	39	7.69	0.08	1.33	0.00	0.10	0.00
	Laniidae	3	100.00	0.00	1.00	0.00	1.00	0.00
	Mimidae	32	3.13	0.06	1.00	0.00	0.03	15.93
	Paridae	6	0.00	0.00	0.00	0.00	0.00	0.00
	Parulidae	224	11.16	0.04	4.92	0.98	0.55	0.38
	Passerellidae	83	12.05	0.07	6.60	1.61	0.80	0.70
	Passeridae	7	0.00	0.00	0.00	0.00	0.00	0.00
	Regulidae	3	0.00	0.00	0.00	0.00	0.00	0.00
	Sturnidae	3	33.33	0.53	3.00	0.00	1.00	0.65
	Troglodytidae	6	0.00	0.00	0.00	0.00	0.00	0.00
	Turdidae	81	17.28	0.08	8.43	0.85	1.46	0.60
	Tyrannidae	9	0.00	0.00	0.00	0.00	0.00	0.00
	Vireonidae	29	0.00	0.00	0.00	0.00	0.00	0.00
**Piciformes**								
	Picidae	21	9.52	0.13	16.50	6.20	1.57	4.35
**Strigiformes**								
	Strigidae	10	50.00	0.31	87.40	151.36	43.70	93.16

Live-caught birds were captured using mist nets, box traps, or spring net traps (USGS Bird Banding Permit # 23877, Arkansas Game and Fish Commission Scientific Collection Permit # 012120221, A-State IACUC # FY20-21-272). Birds were weighed and measured for wing and tail length, then identified to species, sex, and age. To remove lice, we applied a 0.1% pyrethrin-based flea powder (Zodiac, Schaumberg, IL, USA) to lightly cover the wings, sternum, and rump feathers. This powder was placed on the birds a few minutes before ruffling the feathers over a white sheet of paper. The flea powder hyper-activates then paralyzes the lice, which fall to the paper when dislodged by ruffling. This method is a standard protocol for collecting and was supported by Clayton and Drown (2001) as an efficient method to remove lice from live birds. The wing and tail feathers were visually inspected for mites. Finally, the birds were banded with a unique banding code and released. Each host was thoroughly searched for all ectoparasites; however, variation in collecting methods could result in slightly different values.

After ruffling preserved or live birds, lice and mites were visually detected, collected, and stored in tubes with 95% ethanol and stored in a −80 °C freezer to prevent DNA degradation. The specimens are currently stored in −80 °C freezers at Arkansas State University (A-State) and will be cataloged in the Arkansas Center for Biodiversity Collections (ACBC) at A-State. We identified each louse to genus using the keys in Price et al. (2003) under a Zeiss Stemi508 stereomicroscope (Jena, Germany). Voucher images were taken of specimens used for DNA extractions. Host associations were checked using Price et al. (2003) and Smith et al. (2022).

### 2.2 Statistical analysis

We calculated prevalence, mean intensity, and mean abundance of ectoparasites based on Bush et al. (1997) and Rózsa et al. (2000). Prevalence of lice, mites, and the co-occurrence of both were calculated by dividing the number of infested hosts by the entire sample size. This describes the proportion of birds with at least one louse and/or mite present. Mean intensity of lice, or parasite load, was calculated by dividing the total number of lice by only the number of infested hosts. Similarly, mean abundance was calculated by dividing the total number of lice by the total number of hosts, regardless of infestation. We also calculated standard deviation, standard error, and 95% confidence intervals for each statistic using base R in RStudio (v. 4.1.3) (RStudio Team, 2020; R Core Team, 2021) following Bush et al. (1997) and Rózsa et al. (2000). We calculated each statistic at multiple taxonomic levels of bird host, including order, family, and species. We also calculated the statistics for only the birds collected or caught in northeast Arkansas. We statistically compared the prevalence of lice, mites, and thier co-occurrence using a chi-square and Fisher's exact tests (to account for small sample sizes) across all samples and within each host family. We ran all statistical tests in base R in RStudio.

### 2.3 Molecular data

To assess the genetic structure of some lice, we sequenced a portion of the mitochondrial cytochrome oxidase subunit 1 (*cox1*) and nuclear elongation factor 1 subunit alpha (*EF1*-α) genes for lice in the genera *Brueelia* and *Myrsidea*. These two genera were the most common among our samples. Both *cox1* and *EF1-α* are commonly used for identifying, assessing genetic diversity, and estimating phylogenetic relationships of lice (Bush et al., 2016; Madrid et al., 2020). Prior to DNA extraction, voucher images of the dorsal and ventral sides of lice were taken for each individual louse. Our samples included 22 *Myrsidea* spp. from five different host species and 17 *Brueelia* spp. from nine host species (Table S2). We extracted DNA from each individual louse using a modified protocol with a Qiagen QIAamp DNA Micro kit (Qiagen, Valencia, California, USA). After adding Proteinase K, we incubated the lice at 55 °C for ∼48 h before following the standard extraction protocol, similar to Sweet et al. (2018).

We amplified *cox1* and *EF1-α* with polymerase chain reactions (PCR) using a New England Biolabs One Taq Quick-load buffer (Ipswich, Massachusetts). We used the primers H7005 and L6626 for *cox1* (Hafner et al., 1994) and Ef3-for and Cho-10 for *EF1-*α (Danforth and Ji, 1998). Both primer sets started PCR at 94 °C for 2 min with a hot start. This was followed by a temperature sequence of 94 °C for 30 s, then step-up annealing temperatures of 46, 48, 50, and an extension period at 74 °C lasting 30 s. These steps were run for 35 cycles. We ran the PCRs on a SimpliAmp ThermoFisher Thermal cycler (model number A24811). Following PCR, samples were run on a 1% agarose electrophoresis gel to verify amplification. The final PCR products were cleaned with ExoSAP-IT Express (Ipswich, Massachusetts), following the manufacture's protocol. These final products were sent to GENEWIZ (Azenta Life Sciences, South Plainfield, NJ) for Sanger sequencing. We assembled, trimmed, and checked resulting Sanger reads using Unipro UGENE (v. 42.0) (Okonechnikov et al., 2012) to edit misidentified nucleotides and decrease noise along the leading and trailing edges.

### 2.4 Phylogenetic analysis

In addition to our novel sequences, we included existing sequences of *cox1* and *EF1-*α from *Brueelia* (18 samples from Bush et al., (2016) and Sweet et al., (2018)) and *Myrsidea* (18 samples from Kolencik et al., (2022)) from GenBank (Table S2). We also included sequences of outgroup taxa to root our phylogenetic trees: *Theresiella* sp. from Brehm's tiger parrot (*Psittacella brehmii*) for *Brueelia* and *Dennyus* sp. from Chimney swift (*Chaetura pelagica*) for *Myrsidea* (Table S2). We chose the outgroups to be consistent with previous phylogenetic studies of *Brueelia* and *Myrsidea* (Bush et al., 2016; Kolencik et al., 2022). For each genus, we aligned each gene using MAFFT (v.7.471) with the automatic parameter and checked the sequences manually using Seaview (v.5.0.5) (Gouy et al., 2010). We then estimated a maximum likelihood phylogeny for the *cox1* and *EF1*-α genes using IQ-Tree (v.2.1.3) with the MFP function to test for optimal substitution models with ModelFinder (Kalyaanamoorthy et al., 2017) and 100 traditional bootstrap replicates. In addition, we concatenated the two genes for *Brueelia* and *Myrsidea* and ran a partitioned analysis in IQ-Tree using the MFP + MERGE option to test for the optimal substitution models and partitioning scheme with the Bayesian Information Criterion (BIC**) (**Chernomor et al., 2016). We once again used 100 traditional bootstrap replicates. Phylogenies for the *cox1*, *EF1*-α, and concatenated genes for each genus were visualized, rooted, and ladderized in Figtree (v. 2.1.3) (http://tree.bio.ed.ac.uk/software/figtree/). Additionally, we calculated the uncorrected pairwise p-distances for the *cox1* gene among each genus of louse using the ape package (v. 5.6–2) in R (Paradis and Schliep, 2019).

## 3 Results

### 3.1 Diversity and novel host associations of lice

**Fig. 1:**
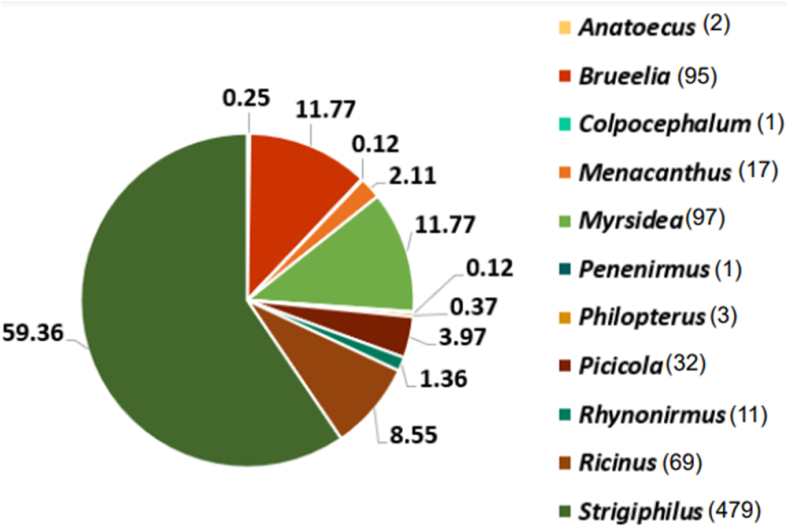
The diversity of louse genera collected from 28 families of birds. Colors associated with each louse genus are indicated in the right-side legend. The numerical values indicate percentages. The sample size is indicated by the parenthesize to the right of genus names. (For interpretation of the references to colour in this figure legend, the reader is referred to the Web version of this article.)

From the 680 birds, we found a total of 817 lice representing 11 different louse genera, including from three different families in the parvorders Ischnocera and Amblycera. Lice collected in Arkansas alone contributed 327 lice. Notably, we collected over half (426) of the 817 lice (all in the genus *Strigiphilus*) from one individual snowy owl (*Bubo scandiacus*) salvaged from Illinois. Lice from songbirds accounted for 276 lice from 21 bird host species. Among songbirds, *Myrsidea* and *Brueelia* were the most common genera of lice with 97 and 95 individuals, respectfully. *Myrsidea* also had the highest number of occurrences, with samples from 21 different host individuals from five host species (Table 1, Table S1, Fig. 1). We found *Brueelia* from 20 different individuals from nine host species (Table S1, Fig. 1). We also found 63 lice in the genus *Ricinus* and 20 lice in the genus *Menacanthus*, both from seven host species (Table S3). Less frequent genera of lice included *Picicola* and *Penenirmus* from a Northern Flicker (*Colaptes auratus*) and Red-bellied Woodpecker (*Melanerpes carolinus*), *Colpocephalum* from Red-tailed Hawk (*Buteo jamaicensis*), and *Rallicola* from Sora (*Porzana carolina*) (Table S3). We also found four novel parasite-host associations not listed by Price et al. (2003) or reported by other checklists. This included *Brueelia* sp. from White-throated Sparrow (*Zonotrichia albicollis*), Lincoln's Sparrow (*Melospiza lincolnii*) and Ovenbird (*Seiurus aurocapilla),* and *Menacanthus* sp. from Tennessee Warbler (*Leiothlypis peregrina*) (Table S3). These lice were all collected from birds in Jonesboro, AR, except for *Menacanthus* sp. from *L. peregrina* collected in Rock Island, IL.

### 3.2 Prevalence, incidence, and abundance of ectosymbionts

The prevalence of lice from birds collected in Arkansas was 7.31% ± 0.028. The largest sample size of Arkansas birds was from Parulidae (Table S4). Parulidae (sample size 109) had a louse prevalence of 6.42% ± 0.05% with a mean intensity of 13.71 and abundance of 0.88. Passerellidae also had a large sample size (37 birds) with a louse prevalence of 13.51% ± 0.11%, mean intensity of 9.20, and abundance of 1.24 (Table S4).

**Fig. 2:**
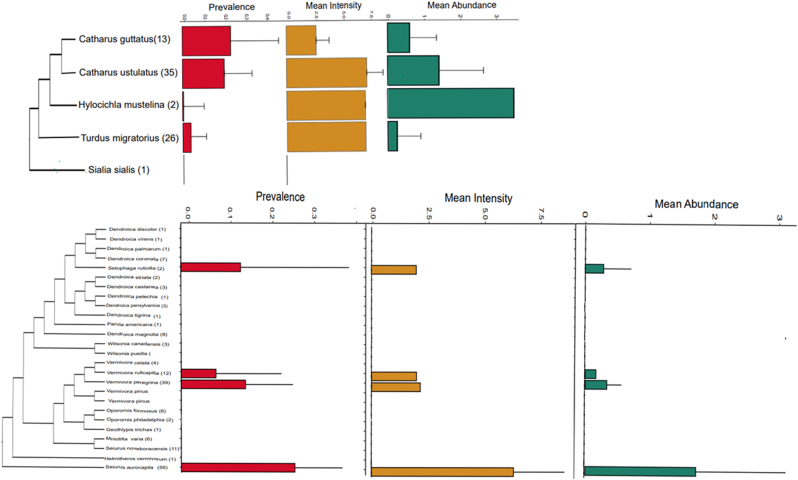
Prevalence, mean intensity, and mean abundance among lice from hosts in the family Turdidae (a) and Parulidae (b). Lines on the bar plots indicate 95% confidence intervals. Parentheses next to species names indicate sample sizes. Phylogenies are cladograms generated from distributions of trees from birdtree.org.

Overall, we collected lice from 72 birds out of 680 sampled individuals, resulting in a 10.57% ± 0.023 prevalence. In total, we found lice from 7 orders and 15 families of birds (Table 1, Table S1). Orders with larger sample sizes (10 or more) with the highest prevalence of lice were owls (Strigiformes) (50%) and songbirds (Passeriformes) (10.25%) (Table 1). We found orders Apodiformes (hummingbirds) and Columbiformes (doves) to have the lowest prevalence (Table 1). Orders Accipitriformes and Anseriformes had high prevalence, but low sample sizes (<3) (Table 1). The highest prevalence of lice among families of songbirds occurred on thrushes (Turdidae) (17.28% ± 0.08%), sparrows (Passerellidae) (12.05% ± 0.07%), and warblers (Parulidae) (11.16% ± 0.04%) (Table 1). Turdidae species with the highest prevalence were Swainson's Thrush (*Catharus ustulatus*) and Hermit Thrush (*Catharus guttatus*) (Table S1, Fig. 2a). We collected no lice from most species in Parulidae with five exceptions: Nashville Warbler *(Leiothlypis ruficapilla)*, Prothonotary Warbler (*Protonotaria citrea*), American Redstart (*Setophaga ruticilla*), *L. peregrina*, and *S. aurocapilla* (Table S1, Fig. 2b). Of these, *S*. *aurocapilla* had the highest prevalence (27.1% ± 0.13%) (Table S1, Fig. 2b).

Mean intensity and abundance of lice also varied among different host taxa. We found Strigidae (87.4% ± 151.36%), Picidae (16.5 ± 6.20%), and Passerellidae (6.6% ± 1.61%) to have the highest mean intensity from families with larger sample sizes (Table 1). Families with the lowest intensity of lice were Icteidae (1.33% ± 0) and Cardinalidae (2.33% ± 0.51%) (Table 1). Among Parulidae, P. *citrea* (100% ± 0) and *S. aurocapilla* (6.31% ± 2.26%) had the highest louse intensity whereas *S. ruticilla* (2.0% ± 0) and *L. ruficapilla* (2.0% ± 0) had the lowest intensities on Parulidae (Table S1; Fig. 2b). The highest mean intensity among species of Turdidae were from American Robin (*Turdus migratorius*) (7.0% ± 0), Wood Thrushes (*Hylocichla mustelina*) (7.0% ± 0), and *C. ustulatus* (6.25% ± 1.15%) (Table S1; Fig. 2a). The lowest intensity among Turdidae was *C. guttatus* (2.67% ± 1.45%) (Table S1; Fig. 2a). Families with the highest mean abundances of lice were Strigidae (43.70% ± 93.17%), Picidae (1.57% ± 4.35%), and Turdidae (1.46% ± 0.60%) (Table 1). Families with the lowest mean abundance were Mimidae (0.03% ± 15.93%), Icteridae (0.10% ± 0) and Cardinalidae (0.21% ± 0.27%) (Table 1). The highest mean abundance in Parulidae were also on P. *citrea* (100%) and *S. aurocapilla* (1.71% ± 1.39%). Mean abundance was lowest on *L. ruficapilla* (0.17% ± 0) and *S. ruticilla* (0.29% ± 0.43%) (Table S1; Fig. 2b). Turdidae with the highest mean abundances were *C. ustulatus* (1.42% ± 1.04%) and *C. guttatus* (0.61% ± 0.75%), whereas *T. migratorius* (0.26% ± 0.65%) had the lowest (Table S1; Fig. 2a).

### 3.3 Co-occurrence of ectosymbionts

**Fig. 3:**
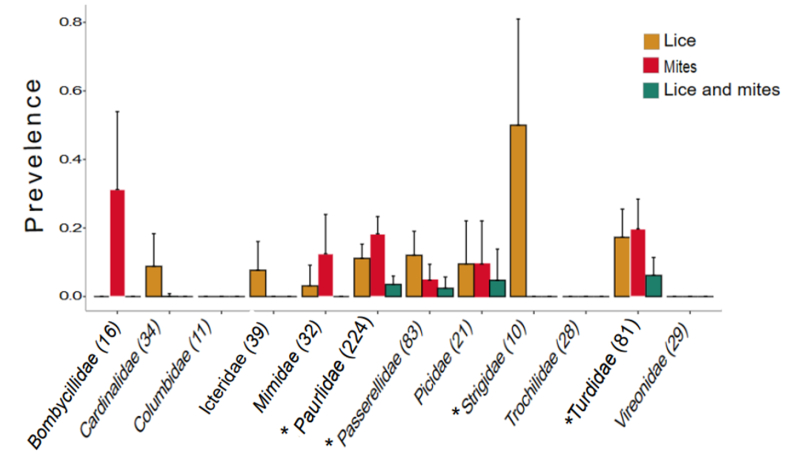
Prevalence of lice, prevalence of mites, and co-occurrence of lice and mites recovered from different families of birds. Parentheses next to family names indicate sample sizes and lines on the bar plots indicate standard error. Significant p-values for chi-square and Fisher's exact tests are indicated by the asterisk to the left of family names.

We found mites from 68 host individuals (9.98% prevalence) from 3 orders and 12 families of birds. Passeriformes had the highest prevalence of mites at 14.43%, followed by woodpeckers (Piciformes) at 9.52% (Table 1). Mites on songbirds occurred most often on Turdidae (20.7%), Passerllidae (3.61%), and Sturnidae (33.33%) (Fig. 3). We found co-occurrence of mites and lice on only 17 birds (2.49%), the majority of which came from four passeriform families (Parulidae, Passerellidae, Picidae, and Turdidae) (Table 2). The highest levels of co-occurrence were on *S. aurocapilla* at 11.86% and *C. ustulatus* at 8.57% (Fig. 3; Table 2). The chi-square and Fisher's exact test indicated there were significant differences among the number of host individuals infested with lice, mites, or co-infestation of lice and mites across all samples (Χ^2^ = 22.631, p = 0.0000121; Fisher p-value = 0.00000245). Host families that also had significant differences in prevalence of lice and mites included Parulidae (Χ^2^ = 46.029, p = 1.01E-10; Fisher p-value = 5.02E-11), Passerellidae (Χ^2^ = 10.1, p = 6.28E-03; Fisher p-value = 1.35E-02), Turdidae (Χ^2^ = 17.3, p = 1.72E-04; Fisher p-value = 7.96E-06), and Strigidae (Χ^2^ = 12, p = 0.002479; Fisher p-value = 0.005305) (Fig. 3). Many of these non-significant values were found among families with lower sample sizes compared to significantly supported families.

**Table 2:** Statistics for prevalence of chewing lice, mites, and co-occurrences sampled from different families of avian hosts with Chi-square and Fisher's exact test p-values.

Order	Family	Sample size	Lice Prevalence	Mite Prevalence	Co-occurrence Prevalence	Chi-square p-value	Fisher's p-value
Accipitriformes							
	Accipitridae	1	100	0	0	Na	Na
Anseriformes							
	Anatidae	3	33.33	0	0	0.223	1
Apodiformes							
	Trochilidae	28	0	0	0	Na	Na
Charadriliformes							
	Scolopacidae	1	100	0	0	Na	Na
Columbiformes							
	Columbidae	11	0	0	0	Na	Na
Gruiformes							
	Rallidae	7	14.285	14.285	0	0.575	1
Passeriformes							
	Bombycillidae	16	0	12.5	0	0.124	0.319
	Cardinalidae	34	8.823	0.058	0	0.229	0.365
	Certhiidae	6	0	0	0	Na	Na
	Corvidae	4	0	0	0	Na	Na
	Emberizidae	3	0	0	0	Na	Na
	Fringillidae	2	0	0	0	Na	Na
	Hirundinidae	1	100	0	0	Na	Na
	Icteridae	39	7.692	0	0	0.046	0.105
	Laniidae	3	100	0	0	Na	Na
	Mimidae	32	3.125	15.625	0	0.024	0.045
	Paridae	6	0	0	3.571	Na	Na
	Parulidae	224	11.16	25	1.204	1.012E-10	5.024E-11
	Passerellidae	83	12.048	3.614	0	0.006	0.014
	Passeridae	7	0	28.571	0	4.421	0.3
	Regulidae	3	0	33.333	0	Na	Na
	Sittiae	2	0	0	0	Na	Na
	Sturnidae	3	33.333	66.666	33.333	Na	Na
	Troglodytidae	6	0	0	0	Na	Na
	Turididae	81	18.181	20.779	6.172	1.71E-4	7.96E-6
	Tyrannidae	9	0	11.111	0	Na	Na
	Vireonidae	29	0	0	0	Na	Na
Piciformes							
	Picidae	21	9.523	9.523	4.761	0.804	1
Strigiformes							
	Strigidae	10	50	0	0	0.002	0.005
							

### 3.4 Phylogeny and genetic distances of *Myrsidea* and *Brueelia* lice

We attempted to amplify 21 *Myrsidea* and 16 *Brueelia* samples. Of these, we successfully sequenced *cox1* from 15 *Myrsidea* and 12 *Brueelia* and *EF1-1α* from 5 *Myrsidea* and 11 *Brueelia*. The *Myrsidea cox1* gene alignment trimmed to 410 base pairs (bp) and *EF1-1α* gene alignment to 413 bp. The *Brueelia cox1* gene alignment trimmed to 420 bp and *EF-1α* gene to 367 bp. The concatenated alignment of both genes was 788 bp with 31 taxa for *Brueelia* and 842 bp with 20 taxa for *Myrsidea*, including outgroups and additional data from GenBank (Table S2).

The most appropriate substitution model for the *cox1* alignment for both *Myrsidea* and *Brueelia* was K3Pu + F + I + G4. The model most appropriate for the *EF-1α* alignment for both *Myrsidea* and *Brueelia* was TNe-R2. The optimal partitioning scheme for the concatenated phylogenies treated the genes as separate partitions for both *Myrsidea* and *Brueelia*.

**Fig. 4:**
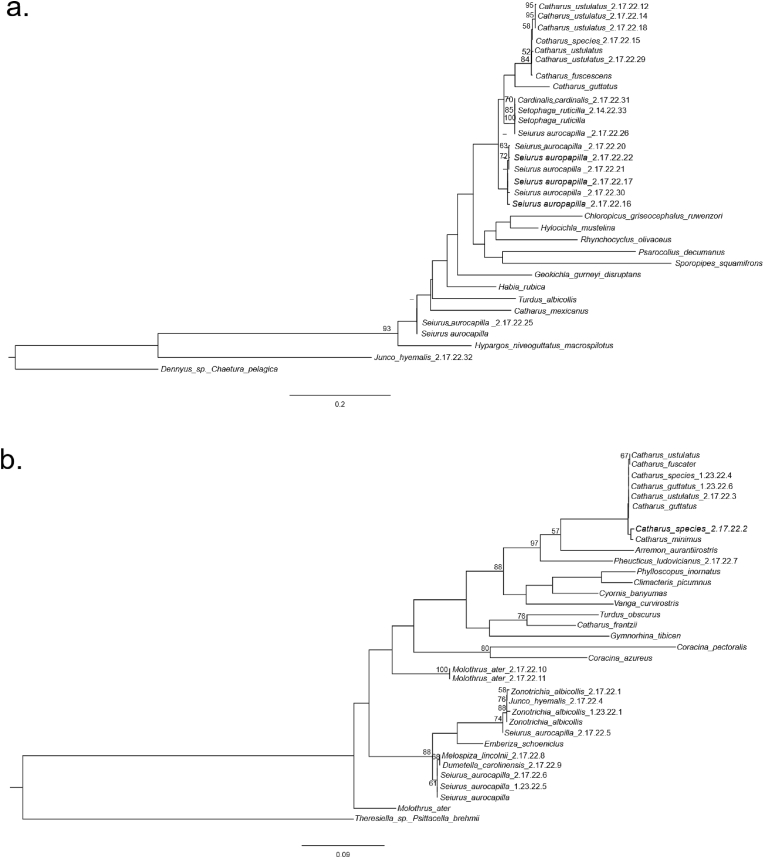
Phylogeny of lice in the genera *Myrsidea* (a) and *Brueelia* (b) based on a concatenated alignment of *cox1* and *EF1-α* sequences. Bootstrap values are located above the associated branches. Only values >50% are shown. Novel samples are labeled with the host species name followed by a 7-digit extraction code. All other ingroup samples were obtained from NCBI GenBank and are labeled with host species names. Outgroups are labeled with genus of louse followed by host species.

The concatenated *Myrsidea* phylogeny had 8 well-supported branches (over 70 bootstrap value) (Fig. 4a). The average *cox1* genetic distance between all *Myrsidea* lice, excluding the outgroup, was 0.1506 (Table S5). The independent *cox1* gene trees had a few notable different phylogenetic relationships compared to the concatenated tree. For example, lice from *S*. *aurocapilla* in the concatenated phylogeny sorted into three clades whereas the *cox1* phylogeny sorted *S*. *aurocapilla* lice into two clades (Fig. 4a, Fig. S1). One *S*. *aurocapilla* clade from the *cox1* tree included only lice from other *S*. *aurocapilla* whereas the other clade included lice from *S. ruticilla* and Northern Cardinal (*Cardinalis cardinalis*). These lice from *S*. *aurocapilla* had an average *cox1* genetic distance of 0.0053 from each other (Table S5). Additionally, the concatenated and *cox1* phylogenies recovered most *Myrsidea* lice from Turdidae in a well-supported group (84 bootstrap) (Fig. 4a, Fig. S1), with an average *cox1* genetic distance of 0.0082 within the clade (Table S5).

The concatenated *Brueelia* phylogeny had 9 well-supported branches with bootstrap values over 70. There were no notable differences between the concatenated and *cox1* phylogenies for *Brueelia* lice. The average *cox1* genetic distance for *Brueelia* lice, excluding the outgroup, was 0.1510 (Table S6). In the concatenated phylogeny, several *Brueelia* lice from Turdidae grouped together in a clade (Fig. 4b; Fig. S6). The average *cox1* genetic distance among these lice was 0.1464 (Table S6). One group (61 bootstrap) of lice included lice from *S. aurocapilla*, *M. lincolnii*, and Gray Catbird (*Dumetella carolinensis)* with an average *cox1* distance of 0.0375 (Table S6; Fig. 4b). Another group included lice from *Z. albicollis,* Dark-eyed Junco (*Junco hyemalis*), and *S. aurocapilla* (74 bootstrap) (Table S6; Fig. 4b). The average *cox1* distance among this clade, excluding the *J. hyemalis* louse without a *cox1* sequence, was 0.0 (Table S6). Similarly, *Brueelia* lice from Brown-headed Cowbird (*Molothrus ater)* came out in an isolated, well-supported group (100 bootstrap) with an average *cox1* distance of 0.0 (Table S6; Fig. 4b). *Molothrus ater* lice from previous studies came out in a separate clade from our *M. ater* lice, with an average *cox1* distance of 0.1364 (Table S6).

## 4 Discussion

### 4.1 Diverse sampling improves our understanding of bird-louse relationships

Sampling deceased and live caught birds allowed us to obtain a large sample size from various bird taxa and habitats in northeastern Arkansas and surrounding states. Although most host associations and louse genera we found were not unexpected, these findings provide useful records for bird lice in the southeastern U.S. Our results are particularly useful for understanding patterns of louse diversity and associations from Passeriformes. For example, lice from the Turdidae species *C. ustulatus* and *C. guttatus* were hosts of lice in the genera *Brueelia*, *Myrsidea*, and *Philopterus*, whereas *T. migratorius* were hosts to only lice in the genus *Ricinus*. It is also notable that *Ricinus* lice are often less abundant on *T. migratorius* compared to *Sturnidoecus* and *Brueelia* based on previous research (Wheeler and Threlfall, 1986; Galloway et al., 2021). Although a larger sample size could reveal other louse taxa that are associated with these species of birds (e.g., *Myrsidea, Menacanthus*, *Sturnidoecus*, and *Brueelia* from *T. migratorius*), our results give unique insight into the regional diversity of lice present on hosts in the southeastern U.S. Furthermore, lice from hosts including hawks (*Colpocephalum*), rails (*Rallicola*), ducks (*Trinoton* and *Anatoecus*), woodcock (*Rhynonirmus*), and woodpeckers (*Picicola* and *Penenirmus*) provide useful records on louse diversity and host associations, although the prevalence, incidence, and abundance of symbionts from these groups should be taken with caution due to lower sample sizes.

The most commonly occurring louse genera from our sample were *Myrsidea* and *Brueelia*. The majority of these individuals were collected from Passeriformes, the most common group of birds in our sample (Table 1). We expected *Myrsidea* and *Brueelia* to be present on many passeriform species because both are well-known to be associated with songbirds (Price et al., 2003; Bush et al., 2016, Kolencik et al., 2022). These two genera have received relatively considerable attention due to their high species diversity and associations with a diverse group of host species (Vas et al., 2008; Bush et al., 2016; Madrid et al., 2020; Kolencik et al., 2022). *Brueelia* spp. were found on the largest diversity of host families in our sample (Fig. 1). However, *Myrsidea* almost exclusively occurred on Parulidae and Turdidae (Table S3). We also found lice in the genus *Ricinus* and *Menacanthus* on Passeriformes. We tended to find *Ricinus* associated with Parulidae such as *L. ruficapilla*, L. *peregrina*, and *P. citrea*, usually at high intensities (Table 1, Table S1). In comparison, lice in the genus *Menacanthus* were associated with several families, including Passerellidae, Lannidae, Sturnidae, and Parulidae, often at low intensities (Table 1, Table S1). The diverse representation of hosts associated with *Menacanthus* were expected based on previously recorded host associations (Price et al., 2003). The differences in intensity and host diversity patterns could be due to dispersal patterns or life histories of the lice, or host-related factors such as body size, habitat, or behavior (Table 1, Table S1) (Clayton and Walther, 2001; Carrillo et al., 2007; Tomás et al., 2016; Galloway and Lamb, 2017).

### 4.2 Novel host associations of louse genera

We found four novel associations between lice (to genus level) and their avian hosts (Table S3). This number of unreported relationships was not surprising due to the lack of research on diversity of bird lice in the southeastern U.S. These novel associations were most commonly found among abundant and well-researched birds such as Passerellidae and Parulidae, which have relatively underreported ectoparasitic data (Price et al., 2003; Bush et al., 2016; Pistone et al., 2021). In most cases the associations were not completely unexpected because of similar lice associated with related host species (Price et al., 2003). However, some new records involved lice from parvorders not recorded for a particular host taxon (Price et al., 2003). For example, we found *Brueelia* on *Z. albicollis, M. lincolnii,* and *S. aurocapilla* hosts. We also commonly found *Menacanthus* lice on *L. peregrina* although not previously recorded across all known hosts association checklist. Although to our knowledge these relationships have never been reported, *Brueelia* and *Menacanthus* are associated with several other closely related species in Passerellidae and Parulidae such as *Melospiza georgiana*, *Passerella iliaca*, and *Dendroica petechia* (Price et al., 2003; Smith et al. 2022).

### 4.3 High variation in prevalence, intensity, and abundance of lice among host taxa

Birds collected from AR had a slightly lower louse prevalence (7.31%) compared to the louse prevalence of all birds sampled (10.57%). The overall prevalence from our study is comparable to the numbers reported from Texas in a 2021 study across different host taxa (13.5%) (Pistone et al., 2021). The decreased prevalence in our study could be related to possible differences in habitat or climate, such as humidity. Lice usually have higher prevalence in humid environments; however, they are also able to survive in arid conditions (Carrillo et al., 2007). Furthermore, most samples from this study were collected from window strikes during migration. During migration, ectoparasite prevalence often increases during winter stayovers in tropical environments but decreases throughout migration to colder climates which could impact the prevalence found from these birds (D'Amico et al., 2008; Sychra et al., 2011). For example, many of the highly sampled species such as S. *aurocapilla, C. guttatus, C. ustalas,* and *D. carolinensis* are non-resident birds in AR. Furthermore, although birds in poor condition were removed from the sample, lice on birds could have left the hosts prior to ruffling.

In general, louse prevalence varies considerably across different host taxa (Table 1, Table S1). Variation among host orders could be due to the wide variety of morphology and habitats across hosts taxonomic groups (Rózsa, 1997; Tomás et al., 2016; Lamb and Galloway, 2019). For example, louse prevalence is often higher on larger hosts compared to smaller hosts (Rózsa, 1997; Galloway and Lamb, 2017). In our sample, louse prevalence for owls was 50% whereas woodpeckers had 9.52%, and none of the *Vireonidae*, *Tyrannidae*, *Regulidae*, and *Trochilidae* in our sample had lice (Table 1). This trend could be due to the abundance of resources and shelter that increases survival on larger hosts (Cotgreave and Clayton, 1994; Galloway and Lamb, 2017). However, many of these host groups had low sample sizes (often <5 individuals), so results should be taken with caution.

Host habitat use or behavior may also influence the prevalence of lice (Tomás et al., 2016). For example, duck lice may have more dispersal opportunities due to their hosts forming large, often mixed species flocks during the winter (Tomás et al., 2016). We also found that songbird hosts which tend to occupy understory habitats, such as *Z. albicollis, S. aurocapilla*, *D. carolinensis*, and several Turdidae species, often had higher louse prevalence compared to Passeriformes that primarily occupy mid or upper canopy (Table 1). This could be attributed to close contact with individuals in understory habitats which facilitates louse dispersal (Tomás et al., 2016)

The largest variation in louse prevalence was in the Parulidae family. We only found lice on S. *ruticilla*, S. *aurocapilla*, *L. ruficapilla*, *L. peregrina*, and *P. citrea*. However, many of these species had low samples sizes and/or louse prevalence. A notable exception was S. *aurocapilla*. *Seiurus aurocapilla* had a large samples size (59) with an overall prevalence of 27.12% and a mean intensity of 6.31 (Fig. 2b). These records were considerably higher than other Parulidae and comparable to louse prevalence and intensity from species in the thrush family (Turdidae). The high prevalence and intensity of S. *aurocapilla* lice could be due their relatively large body size compared to other parulids or use of understory habitat, which is often more similar to many species in Turdidae than other species of warblers (Sieving and Willson, 1998; King et al., 2006; Sychra et al., 2011; Haché et al., 2017).

### 4.4 Co-occurrence of ectosymbionts

The prevalence of co-occurrence of lice and mites on the same host was lower than the prevalence of each type of ectosymbiont individually. Additionally, co-occurrence was present for only a few hosts families such as Parulidae, Passerellidae, Picidae, and Turdidae (Fig. 3).-. This could be due to competition for resources, associations with hosts' health, or different environmental pressures (Krasnov et al., 2020). Lice and mites both rely on host feathers for shelter and/or food. The mites most often found in this study specifically live on tail or wing feathers and perhaps consume microorganisms on the host's feathers, although the diets of feather mites are still relatively unknown (Dona et al., 2019). Additionally, at high lice intensities, lice may be consuming a large quantity of feathers which could decrease the abundance of food for mites. Furthermore, in the presence of symbiont co-occurrence, lice may be competing with mites for limited space on wing feathers, as seen with certain mite species (Choe and Kim, 1989). Lice, especially lice adapted to wing feathers (“wing lice”), use these feathers to avoid being preened off by their hosts (Bush et al., 2006). The larger size and mobility of lice could be advantageous for lice to use space and lead to a decreased habitat for mites. In the future, this research could be expanded to compare the negative correlation between louse and mite intensity to further support this observation.

Furthermore, the low occurrence of lice and mites on the same host could be due to different relationships with host's health. Lice are often found at high intensities on sick or weak birds that cannot afford to expend energy on preening, whereas mites are not known to be associated with hosts in poor health (Ash, 1960). It is possible co-occurrences are more common on sick birds because lice are more likely to occur. Additionally, the low co-occurrence of lice and mites could also be due to lice predation on mites; however, this has not been observed. Alternatively, the patterns of prevalence between mites and lice could be related to seasonal variation, age, or species of mites, which were not identified in the present study (McClure, 1989). Cryptic diversity among parasites can be difficult to identify even at the genus level, but future studies should focus on identifying and comparing particular taxa of mites and lice species to further understand this variation in prevalence.

It is notable that there was also a low co-occurrence of multiple lice genera on individual hosts. Additionally, on hosts with multiple genera, intensity of at least one genus was usually much lower. For example, lice in the genus *Ricinus* had a mean intensity of 6.25 when found alone, however in the single instance when co-occurring with other louse genera, mean intensity decreased to 1.0 (i.e., we only found a single *Ricinus* individual). Another notable occurrence was the louse genus *Philopterus*, which was only found on hosts that also had *Myrsidea* or *Brueelia* lice. The low occurrence of multiple lice genera on a single host could also be related to competition for limited resources, hosts lifestyles, or predation.

### 4.5 Phylogenetic patterns of lice reflect host taxonomy and ecology

Based on the concatenated gene phylogenies of *Myrsidea* and *Brueelia*, these lice typically have close evolutionary relationships to lice from similar host taxonomic groups or shared habitats. Lice often rely on specific host morphology, such as feather and body size, for survival (Johnson et al., 2005). These host traits are often shared among species in the same taxonomic family or order. For example, our samples of *Brueelia* and *Myrsidea* lice from Turdidae were in monophyletic groups with other Turdidae lice. This is possibly due to similar host body sizes and habitats which allow lice to disperse and survive on multiple thrush species. Lice primarily rely on direct contact between hosts for dispersal (Rothschild and Clay 1952; Clayton and Tompkins, 1994; Brooke, 2010). Because interactions are often higher among individuals in shared habitats, those could increase opportunity for distantly related hosts to share parasites (Brooke, 2010). For example, a *Brueelia* louse from *D. carolinensis* was grouped together with lice from several species of Passerellidae and *S. aurocapilla*. Although *D. carolinensis* is not closely related or morphologically similar to Passerellidae or Parulidae, this relationship could have occurred because of the shared understory habitat between these hosts. Similarly, *Brueelia* lice from Passerellidae and *S. aurocapilla* were clustered together. Although these two hosts are not closely related, the relationships among their lice might occur from utilizing similar habitats, which could increase the likelihood of lice dispersing between the two hosts. Furthermore, *Myrsidea* lice from *S. aurocapilla*, *C. cardinalis* and *S. ruticilla* were sorted together despite their distantly related hosts. This mixed group of hosts could also be related to the shared habitat of these species (Burke and Nol, 1998; Leston and Rodewald, 2006; Donajkowski, 2009).

Although host taxonomy and habitat as drivers of louse evolutionary relationships were supported by several examples, there were some notable exceptions. For example, in the *Myrsidea* phylogeny two lice from *S. aurocapilla*, including a louse from a previous study, were distantly related to the other *S. aurocapilla* lice. The genetic separation of these lice could reflect morphologically ambiguous yet reproductively isolated cryptic species. We found a similar pattern in some *Brueelia* lice from *M. ater*. Our two samples of *M. ater* lice were grouped together but were distantly related to *M. ater* lice from a previous study. This relationship could be related to the brood parasitism strategy of *M. ater*. Brood parasites rely on non-related individuals, usually of different species, to raise offspring of the brood parasite (Servedio and Hauber, 2006). This type of interaction could allow lice from the host parent to transfer lice to *M. ater*. Furthermore, the decrease in louse transfers among familial *M. ater* could decrease gene flow among lice and allow species divergence of the lice. Although *M. ater* have particular species of lice that are acquired from interactions with other *M. ater*, our results indicate a possible unrecorded association or cryptic diversity from either a parasitized or *M. ater* louse species (Clayton and Johnson, 2001). Future work should rely on morphological characters to rigorously assess the taxonomy of potentially cryptic species identified in our phylogeny.

## 5 Conclusion

Through a combination of approaches, we surveyed prevalence, intensity, abundance, and host relationships of avian chewing lice in northeastern Arkansas. Because little research has analyzed chewing lice in the southeastern United States, this study will provide useful data for future parasite research, such as studies focused on changes in prevalence of lice or host associations over time or location. Overall, we found a diverse assortment of chewing lice from many hosts species, including several new louse-host associations. Additionally, we found that the prevalence of lice can vary among different host taxa. We also found a low co-occurrence of mites and lice on the same host individual. Our results further support that the associations between lice and their hosts are often structured by host ecology and/or taxonomy.

## Funding

This work was supported by a Student Undergraduate Research Fellowship (SURF) from the Arkansas Department of Higher Education to PJB, and by 10.13039/100008231Arkansas Biosciences Institute (ABI) Seed grant # 21-128-21 to ADS.

## Author’s contributions

Conceptualization: ADS; Data Curation: PJB; Formal Analysis: PJB, ADS; Funding acquisition: PJB, ADS; Methodology: PJB, ADS; Supervision: ADS; Visualization: PJB; Writing – original draft: PJB; Writing – review and editing: PJB, ADS.

## Data availability

All data are available as supplementary material or in Figshare (https://doi.org/10.6084/m9.figshare.23688954.v1 https://doi.org/10.6084/m9.figshare.23688900.v1 https://doi.org/10.6084/m9.figshare.23688666.v1 https://doi.org/10.6084/m9.figshare.23688954.v1). Novel sequence data are available on NCBI GenBank. The genbank numbers are 2735735, 2728807, and OR460840 - OR460853.

## Declaration of competing interest

The authors declare they have no competing interests.
