# Supplementary material for: Prevalence and diversity of parasitic bird lice (Insecta: Psocodea) in northeast Arkansas
Source: Int J Parasitol Parasites Wildl. 2023 Jul 21;22:205–15. doi: 10.1016/j.ijppaw.2023.06.007 (PMC10628595; doi:10.1016/j.ijppaw.2023.06.007)
Supplement: Multimedia component 1 [file mmc1.docx]

Supplemental figures captions:

Figure S1. Phylogeny of lice in the genus *Myrsidea* using the mitochondrial *cox1* gene. Bootstrap values over 50% are shown on the left side of the nodes. Tips are labeled with hosts species name and 7-digit extraction code.

Figure S2. Phylogeny of lice in the genus *Brueelia* using the mitochondrial *cox1* gene. Bootstrap values over 50% are shown on the left side of the nodes. Tips are labeled with hosts species name and 7-digit extraction code.

Table S1. Lice prevalence, mean intensity, and mean abundance with 95% confidence intervals among all families and species of birds collected.

Table S2. Genetic sampling of *cox1* and *efa1-a* of *Myrsidea* and *Brueelia*. NCBI GenBank accession numbers are listed.

Table S3. Diversity and quantity of lice genera, with presence/absence of mites for each host infested with at least one louse.

Table S4. Lice prevalence, mean abundance, and mean intensity with 95% confidence intervals among family and species of hosts of birds collected in only AR.

Table S5. Uncorrected genetic distances from cox1 of *Myrsidea*.

Table S6. Uncorrected genetic distances from cox1 of *Brueelia*.
