# Supplementary figures and images for: Prevalence and diversity of parasitic bird lice (Insecta: Psocodea) in northeast Arkansas
Source: Int J Parasitol Parasites Wildl. 2023 Jul 21;22:205–15. doi: 10.1016/j.ijppaw.2023.06.007 (PMC10628595; doi:10.1016/j.ijppaw.2023.06.007)

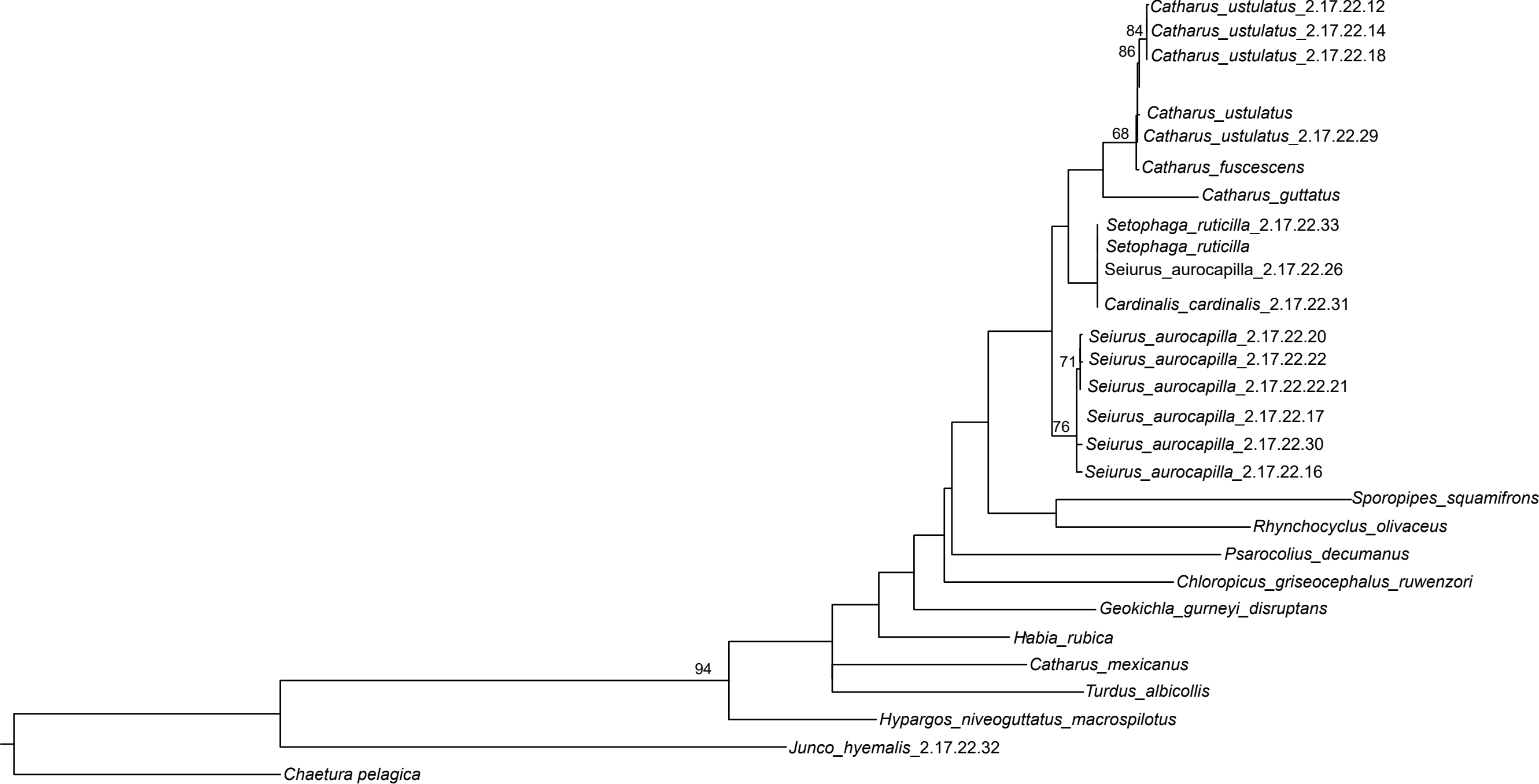

Supplement: Multimedia component 2 [file mmc2.pdf]

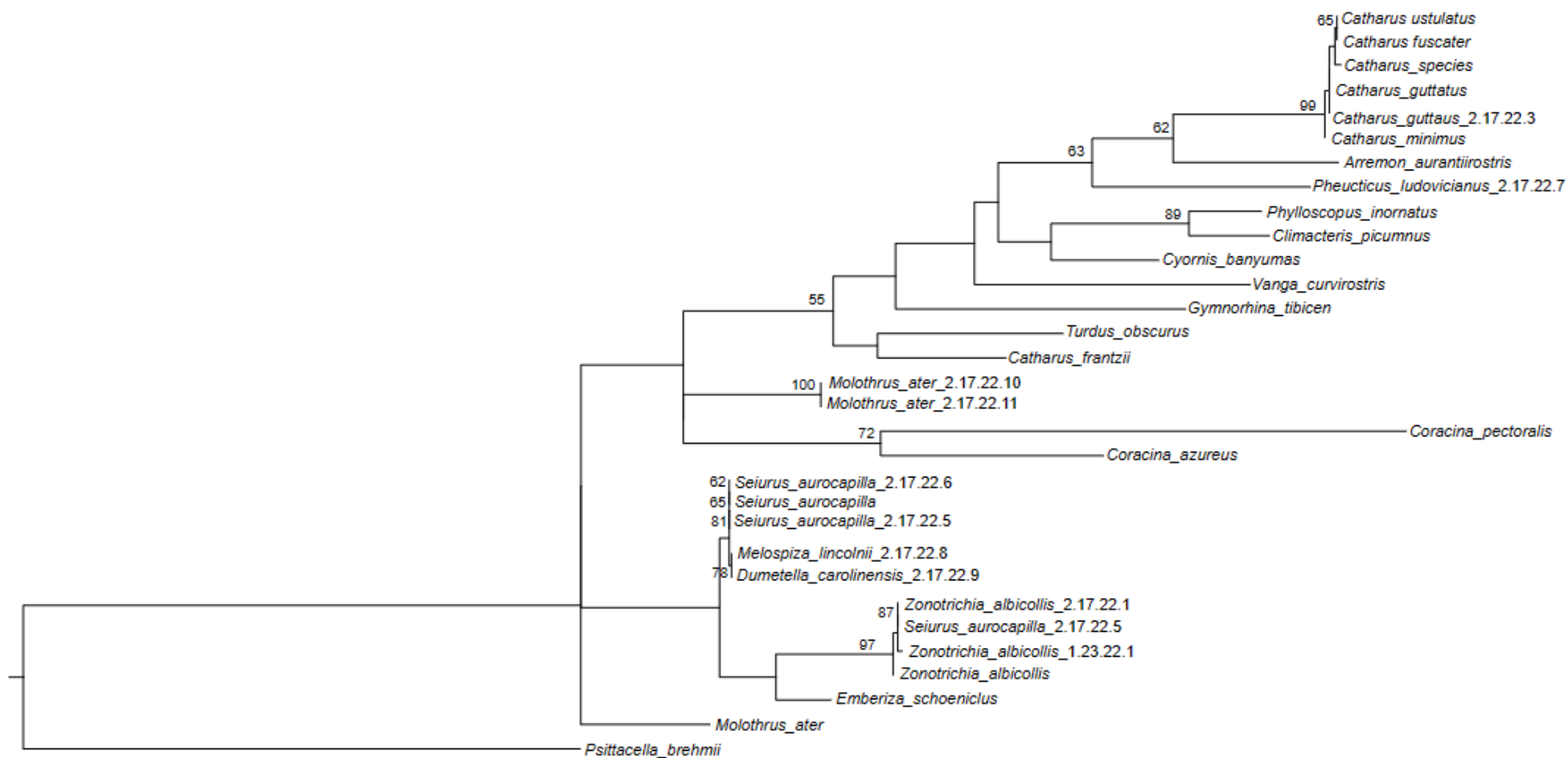

0.2

Supplement: Multimedia component 4 [file mmc4.pdf]
